# National Income Inequality and Declining GDP Growth Rates Are Associated with Increases in HIV Diagnoses among People Who Inject Drugs in Europe: A Panel Data Analysis

**DOI:** 10.1371/journal.pone.0122367

**Published:** 2015-04-15

**Authors:** Georgios K. Nikolopoulos, Anastasios Fotiou, Eleftheria Kanavou, Clive Richardson, Marios Detsis, Anastasia Pharris, Jonathan E. Suk, Jan C. Semenza, Claudia Costa-Storti, Dimitrios Paraskevis, Vana Sypsa, Melpomeni-Minerva Malliori, Samuel R. Friedman, Angelos Hatzakis

**Affiliations:** 1 Department of Hygiene, Epidemiology and Medical Statistics, Athens University Medical School, Athens, Greece; 2 Hellenic Centre for Disease Control and Prevention, Amarousio, Greece; 3 Greek Reitox Focal Point of the EMCDDA, University Mental Health Research Institute, Athens, Greece; 4 Department of Psychiatry, Athens University Medical School, Athens, Greece; 5 Panteion University of Social and Political Sciences, Athens, Greece; 6 European Centre for Disease Prevention and Control (ECDC), Stockholm, Sweden; 7 European Monitoring Centre for Drugs and Drug Addiction (EMCDDA), Lisbon, Portugal; 8 National Development and Research Institutes, New York, New York, United States of America; University of South Carolina, UNITED STATES

## Abstract

**Background:**

There is sparse evidence that demonstrates the association between macro-environmental processes and drug-related HIV epidemics. The present study explores the relationship between economic, socio-economic, policy and structural indicators, and increases in reported HIV infections among people who inject drugs (PWID) in the European Economic Area (EEA).

**Methods:**

We used panel data (2003–2012) for 30 EEA countries. Statistical analyses included logistic regression models. The dependent variable was taking value 1 if there was an outbreak (significant increase in the national rate of HIV diagnoses in PWID) and 0 otherwise. Explanatory variables included the growth rate of Gross Domestic Product (GDP), the share of the population that is at risk for poverty, the unemployment rate, the Eurostat S80/S20 ratio, the Gini coefficient, the per capita government expenditure on health and social protection, and variables on drug control policy and drug-using population sizes. Lags of one to three years were investigated.

**Findings:**

In multivariable analyses, using two-year lagged values, we found that a 1% increase of GDP was associated with approximately 30% reduction in the odds of an HIV outbreak. In GDP-adjusted analyses with three-year lagged values, the effect of the national income inequality on the likelihood of an HIV outbreak was significant [S80/S20 Odds Ratio (OR) = 3.89; 95% Confidence Interval (CI): 1.15 to 13.13]. Generally, the multivariable analyses produced similar results across three time lags tested.

**Interpretation:**

Given the limitations of ecological research, we found that declining economic growth and increasing national income inequality were associated with an elevated probability of a large increase in the number of HIV diagnoses among PWID in EEA countries during the last decade. HIV prevention may be more effective if developed within national and European-level policy contexts that promote income equality, especially among vulnerable groups.

## Introduction

The annual numbers of newly diagnosed HIV cases among people who inject drugs (PWID) varied across European countries during the ten-year period between 2003 and 2012. Most countries have experienced either stable (e.g., Croatia, Cyprus, the Czech Republic, Norway, Slovenia, Slovakia,) or declining (e.g., Austria, Belgium, France, Ireland, Netherlands, Poland, Portugal, United Kingdom) trends in the reported numbers of new HIV diagnoses.[1,2] Some countries (e.g., Lithuania, Latvia, Spain, and Sweden) had increases which, nonetheless, were not sustained more than 1–2 years. On the other hand, two European Union (EU) countries, Greece and Romania, experienced large HIV outbreaks in 2011 and 2012, [3,4] while rising numbers have also been observed in Bulgaria since 2006.[5]

The recent HIV epidemics in PWID appeared amidst economic recession and in countries, especially in Greece, which were seriously affected by the global financial crisis.[6] Rapid and wide-scale spread of HIV among PWID has been shown to have occurred in the context of big economic changes. For instance, high levels of HIV transmission coincided with or followed severe social, political, and economic disruption in Russia and other former Soviet Union states in the early 1990s.[7,8] It should be noted that the coverage of opioid substitution treatment (OST) and needle and syringe programs (NSP) had been constantly low in Greece before the outbreak. The estimated OST coverage was about 28% in 2010, while NSPs were distributing annually below 20 sterile syringes per injecting drug user [9]—well below the international standards. In Romania, the interruption of international funding disrupted prevention services. In 2011, approximately 10% of those in need were in OST while the NSP coverage in Bucharest, the capital city, was 46 syringes per injector per year.[10]

The effects of economic downturn on population morbidity and mortality,[11] including infectious diseases,[12,13] have attracted considerable scientific interest. The spread of HIV among PWID is likely to be the result of a complex interplay between various factors that affect the probability of HIV acquisition.[14,15] Although many models have been proposed to describe the complexity of HIV transmission,[7,15] we can discern three categories of factors that may be involved in the process: 1) macro-level parameters or distal causes such as “Big Events” (wars, economic downturn, and transitions) [7] and other macro-level factors such as income inequality;[16] 2) policy-level factors or intermediate causes such as governmental expenditures on health and social protection, the availability of HIV prevention measures including OST and NSP,[14,17–19] and the presence of drug control policies and punitive environments leading to incarceration;[20] and 3) factors associated with injecting and sexual practices or proximal causes such as receptive sharing of injection paraphernalia, injecting frequency and history, use of stimulants, and unprotected sex.[14]

Despite extensive discussion on the health consequences of economic instability, there is sparse quantitative evidence on the ecological relationships between drug-related HIV epidemics and population-level parameters in Europe.[15,21] Therefore, the present study aims to evaluate the association between economic, social, and other related variables and increases observed in the HIV diagnosis rate among PWID in European Economic Area (EEA) countries during a period of economic upheaval.

## Methods

### Data collection

The primary outcome measure (probability of an HIV outbreak in PWID) was based on the annual numbers of newly diagnosed HIV cases attributed to injecting drug use in 30 EEA countries (2003–2012). HIV data were obtained from the European Surveillance System (TESSy) of the European Centre for Disease Prevention and Control (ECDC). The outbreak was defined as a statistically significant increase in the annually reported HIV diagnoses among drug injectors. The explanatory variables (Table 1) were selected based on theoretical relevance and their availability. They consisted of: 1) macro-level parameters including indicators of countries’ wealth—e.g., Gross Domestic Product (GDP) per capita and GDP growth rate, socio-economic indicators—e.g., the share of the population that is at risk of poverty, unemployment rate, national level of income inequality [expressed in terms of Gini coefficient and the ratio of total income received by the 20% of the population with the highest income to that received by the 20% of the population with the lowest income (Eurostat S80/S20 ratio)], and the public wealth index (PWI—the division of the Eurostat GDP per capita by the Eurostat S80/S20 ratio);[22] 2) policy variables such as per capita government expenditure on health and social protection, the number of people receiving opioid substitution treatment and the number of needles/syringes distributed through needle and syringe distribution programs (as proxies for harm reduction coverage), and recorded crimes related to drug trafficking (proxy for drug control policy); and 3) variables related to the drug-using population of each country and its practices including new entries to drug treatment (proxy for the magnitude of the drug problem in a country), the estimated numbers of problem drug and problem injecting drug users (indicators of the magnitude of problem drug use which includes injecting drug use or long duration or regular use of opioids, cocaine and/or amphetamines), injecting drug use, opioid or cocaine injecting, and daily opioid use (all indicators of high-risk injecting behavior). Data on explanatory variables were retrieved from Eurostat and the European Monitoring Centre for Drugs and Drug Addiction (EMCDDA) for the years 2000–2011 (to explore lagged effects).

**Table 1:** Description of variables used in the study.

Variables	Description	Source	Coverage 2002–2011	
			Countries (n)	Observations (n)
**Macro-level**				
GDP per capita	Nominal Gross Domestic Product in Purchasing Power Standards per capita	EUROSTAT	30	300
GDP growth rate	Percentage change of GDP from one year to the next	EUROSTAT	30	300
S80/S20 ratio	Measure of income inequality. The ratio of total income received by the 20% of the population with the highest income (the top quintile) to that received by the 20% of the population with the lowest income (the bottom quintile)	EUROSTAT	30	261
Gini coefficient	Measure of income inequality. It measures the extent to which the distribution of income (or, in some cases, consumption expenditure) among individuals or households within an economy deviates from a perfectly equal distribution. Scale 0–100	EUROSTAT	30	258
Public wealth index (PWI)	GDP per capita divided by the S80/S20 ratio	Suk et al, 2009	30	261
Population at risk of poverty	Share of people with an equivalised disposable income (after social transfer) below the at-risk-of-poverty threshold, which is set at 60% of the national median equivalised disposable income after social transfers	EUROSTAT	30	259
Unemployment	Number of people unemployed as percentage of labor force (%)	EUROSTAT	30	299
**Policy level**				
Government Expenditure: Health	Total general government expenditures on Health (millions of euro)	EUROSTAT	29	290
Government Expenditure: Social protection	Total general government expenditures on Social protection (millions of euro)	EUROSTAT	29	290
Crimes: drug trafficking	Crimes recorded by the police relating to drug trafficking (n)	EUROSTAT	30	268
Syringes distributed or exchanged	Number of syringes provided through Needle and Syringe programmes (n)	EMCDDA	26	158
OST clients	Number of people receiving opioid substitution treatment (OST) (n)	EMCDDA	28	206
**Drug user level**				
Problem drug users^†^	Estimated size of population of problem drug users (rate per 1000 population aged 15–64)	EMCDDA	25	102
Problem injecting drug users^††^	Estimated size of population of problem injecting drug users (rate per 1000 population aged 15–64)	EMCDDA	16	59
New clients entering treatment	Annual number of people entering for first time treatment for drug-related problems (n)	EMCDDA	28	253
Opioid injectors	Percentage of treatment entries with opioids as primary substance who report injection as the main route of administration (%, of all opioid outpatient treatment entries)	EMCDDA	29	166
Cocaine injectors	Percentage of treatment entries with cocaine as primary substance who report injection as the main route of administration (%, of all cocaine outpatient treatment entries)	EMCDDA	29	162
Daily opioid use	Opioid-related treatment entries who report daily use (%, of all opioid outpatient treatment entries)	EMCDDA	29	155
HIV cases among People Who Inject Drugs (PWID)^†††^	HIV case reports with injecting drug use as the probable route of transmission (n)	ECDC	30	284

*Notes*.

^†^ National estimated trends for problem drug users were available by the EMCDDA only for 12 countries from 2006 to 2011 (48 observations, Table PDU-6 PART-I). Missing data for some years and countries were filled-in by combining data from national full lists (Table PDU-102 PART-I);

^††^ National estimated trends for problem injecting drug users were available by the EMCDDA only for seven countries from 2006 to 2011 (34 observations; Table PDU-6 PART-III). Missing data for some years and countries were filled-in by combining data from national full lists (Table PDU-102 PART-II);

^†††^Coverage: 2003–2012.

### Statistical Analysis

We ran log-linear regressions of HIV rates among PWID versus year of report (2003–2012) for each individual country to identify those with statistically significant increases in HIV diagnoses (p < 0.01). The analyses showed that there were three countries in which rates of newly diagnosed HIV cases among PWID had increased significantly: Greece, Romania, and Bulgaria (S1 Table). The outbreak years for Greece and Romania included 2011 and 2012. It was difficult to visually select the start of the increase (between 2006 and 2007) in Bulgaria, which was eventually statistically determined to be 2006. Specifically, we applied two Poisson regressions to Bulgarian data modelling HIV rate as the dependent variable and calendar year as a binary factor (taking value 1 the first year the increase was observed and afterwards, and 0 before HIV rates start to elevate). The estimated beta was bigger and significant when we used 2006 [1.73, 95% Confidence Interval (CI): 1.27 to 2.19] rather than 2007 (1.16, 95% CI: 0.87 to 1.45) as the year the increase began.

The dependent variable for the main analysis was a dichotomous variable that took the value 1 for each year in which a European country experienced an HIV outbreak in PWID during the period 2003–2012 and 0 otherwise. We assumed that unmeasured differences across countries (such as time-invariant policies or cultural characteristics), which are unrelated to the predictors mentioned above, have some influence on the outcome and each country has thus its own probability for an HIV outbreak (different intercepts). Because most European countries experienced no HIV outbreaks (constant outcome over time), it was not possible to employ fixed-effects models. Therefore, the analysis involved random-effects (random intercept) logistic regression models [23]:

$${\text{Pr(y}}_{\text{it}}\neq \text{0|}{\text{x}}_{\text{it}}\text{)}=\text{F(}{\text{x}}_{\text{it}}\beta +\nu_{i})$$

for i = 1,…30 countries, where t = 1,…..10 years (of which some or all are observed), ν_i_ are country-specific random-effects independently, identically and normally distributed {N (0, σ^2^_ν_)}, **x**_**it**_ a vector of regressors (e.g. GDP growth rate), **β** a vector of coefficients estimated via maximum likelihood, Pr the probability of an HIV outbreak in country i at time t given the regressors, and F(z) = {1 + exp(-z)}^-1^. Random-effects approaches are most useful when the objective is to make inferences about individual countries. All country-specific βs in the text and tables are presented as Odds Ratios (OR) and were calculated by exponentiating β_s_.

We initially explored univariable associations between the dependent variable and the predictors. Those variables that reached significance at a level of p < 0.05 were included in multivariable models. We calculated Spearman’s rank correlation coefficients for the pairs of independent variables that entered the multivariable phase. Highly correlated variables (such as Gini coefficient, S80/S20 ratio and the proportion of people at risk for poverty) were not simultaneously inserted in multivariable regression models to minimize multicollinearity effects. To avoid reverse causality and to account for the delayed impact of some explanatory factors, we examined lagged effects (1 to 3 years prior the current observations). All statistical analyses were conducted in Stata 12.0.

## Results

**Fig 1:**
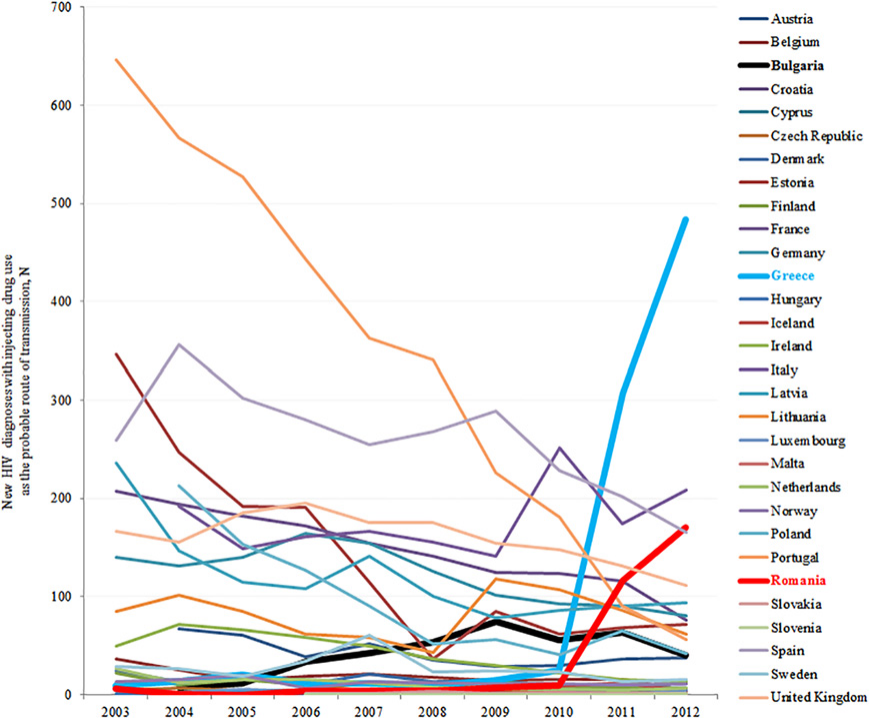
Numbers of new HIV diagnoses with injecting drug use as the probable route of transmission in 30 countries of the European Economic Area (2003–2012). Contrary to the overall trend, Greece and Romania observed large increases in the number of HIV diagnoses among people who inject drugs in 2011. Rising numbers have also been observed in Bulgaria since 2006.

Fig 1 shows the 10-year trends in the number of newly diagnosed HIV cases related to injecting drug use in 30 EEA countries. The 10-year mean European HIV rate among PWID was 10.71 diagnoses per million population. Greece reported the largest increase in new HIV diagnoses among PWID in 2011–2012, followed by Romania, while in Bulgaria the change was less abrupt and started earlier (2006–2012). Totally, 11 observations of these 3 countries represented outbreak years. Table 2 presents summary statistics of the explanatory variables used in the analyses. As shown in Table 1, many policy variables and variables related to the drug using population had multiple missing values and thus could not be included in statistical analyses.

**Table 2:** Summary statistics for explanatory variables (years: 2002–2011).

	Observations	Mean	Std. Dev.	Min	Max	Unit of measurement
Gross Domestic Product (GDP) per capita	300	23.15	10.52	6.00	68.50	Thousands Purchasing Power Standards (PPS), per capita
GDP growth rate	300	2.29	3.93	-17.70	11.20	%
Government Expenditure: Health	290	15.63	11.19	0.72	52.07	Hundreds €, per capita^†††^
Government Expenditure: Social protection	290	40.47	31.10	2.25	150.82	Hundreds €, per capita^†††^
Population at risk of poverty	259	15.39	3.85	8.60	25.70	%
Unemployment	299	8.13	3.82	2.30	21.70	%
S80/S20 ratio	261	4.64	1.12	3.00	7.90	Ratio
Gini coefficient	258	29.16	4.00	22.00	39.20	Scale 0–100
Public wealth index (PWI)	261	5.59	3.05	1.28	17.13	GDP per capita (Thousands PPS) divided by S80/S20
Crimes: drug trafficking	268	7.88	8.73	0.49	51.27	…per 10,000 population^†††^
New clients entering treatment	253	28.33	20.25	0.95	102.96	…per 100,000 population^†††^
Opioid Substitution Treatment (OST) clients	206	0.94	0.81	0.02	2.76	… per 1,000 population^†††^
Syringes distributed or exchanged	158	14.54	21.88	0.00	138.00	n*100,000
Opioid injectors^†^	166	22.70	37.61	0.28	219.92	…per 100,000 population^†††^
Cocaine injectors^†^	162	6.91	17.22	0.00	134.09	…per million population^†††^
Total injectors^††^	150	20.39	28.38	0.47	140.45	…per 100,000 population^†††^
Daily opioid use	155	65.03	17.36	2.20	94.50	%

*Notes*.

^†^ The number of injectors for each primary substance use (opioid and cocaine) was calculated from each annual Table TDI-17 part-ii and part-iv, respectively, by multiplying the relevant percentage by the number of clients whose usual route of administration was known;

^††^ The total number of injectors was obtained by adding the numbers of injectors of each substance (opioid, cocaine, stimulants, and cannabis), calculated from the percentages in the tables;

^†††^All per population estimates were obtained by dividing the actual variable values (derived from EUROSTAT or EMCDDA) by population estimates derived from Eurostat.

### Univariable analyses

Table 3 presents the results of univariable logistic regression models using lagged values of the explanatory variables, i.e., one (Lag1), two (Lag2) and three (Lag3) years before the current observation. The estimated ORs for an HIV outbreak associated with a 1% yearly increase in GDP were 0.78 (95% CI: 0.62 to 0.98), 0.66 (95% CI: 0.49 to 0.88) and 0.81 (95% CI: 0.65 to 1.02) for Lag1, Lag2 and Lag3 observations, respectively. Both of the indicators of inequality in national income distribution were positively related to the odds of an HIV outbreak among drug injectors in EEA countries. In particular, the estimated ORs for an HIV outbreak associated with the per unit increase in the S80/S20 ratio (higher inequality) ranged from 3.07 (95% CI: 1.10 to 8.57) for Lag2 values to 3.82 (95% CI: 1.09 to 13.40) for Lag3 values. The estimates for Gini coefficient reached a similar level of statistical significance. Higher proportions of people at risk for poverty were associated with heightened probability of an HIV outbreak among PWID in EEA countries. In particular, given 1% increase in the share of population that was facing substantial poverty risk, an HIV outbreak was from 63% to 80% more likely depending on the lag. Finally, using 1-year lagged values, the PWI was also significantly associated with the likelihood of an HIV outbreak among drug injectors in EEA countries (OR: 0.28, 95% CI: 0.08 to 0.96 for Lag1 values, OR: 0.37, 95% CI: 0.13 to 1.01 for Lag2 values, and OR: 0.44, 95% CI: 0.18 to 1.11 for Lag3 values).

**Table 3:** Univariable logistic regression models.

	LAG 1 (1 year before current observation)						LAG 2 (2 years before current observation)						LAG 3 (3 years before current observation)					
		95% CI						95% CI						95% CI				
	OR	L	U	*P*	Obs.	C	OR	L	U	*P*	Obs.	C	OR	L	U	*P*	Obs.	C
Gross Domestic Product (GDP) per capita	0.90	0.72	1.12	0.336	300	30	1.02	0.85	1.23	0.829	300	30	1.08	0.91	1.27	0.389	300	30
**GDP growth rate**	**0.78**	0.62	0.98	**0.031**	300	30	**0.66**	0.49	0.88	**0.004**	300	30	**0.81**	0.65	1.02	**0.070**	299	30
Government Expenditure: Health^†^	0.83	0.66	1.06	0.131	290	29	0.90	0.73	1.11	0.337	289	29	0.99	0.81	1.21	0.941	288	29
Government Expenditure: Social protection^†^	0.96	0.88	1.04	0.322	290	29	0.97	0.89	1.05	0.417	289	29	0.97	0.89	1.06	0.490	288	29
**Population at risk of poverty**	**1.80**	1.16	2.78	**0.009**	259	30	**1.63**	1.09	2.42	**0.017**	253	30	**1.69**	1.05	2.72	**0.031**	245	30
Unemployment	0.98	0.74	1.29	0.894	299	30	0.76	0.52	1.11	0.156	298	30	0.48	0.23	1.04	0.062	297	30
**S80/S20 ratio**	**3.13**	1.15	8.53	**0.025**	261	30	**3.07**	1.10	8.57	**0.032**	255	30	**3.82**	1.09	13.40	**0.036**	248	30
**Gini coefficient**	**1.43**	1.03	1.98	**0.033**	258	30	**1.40**	1.03	1.91	**0.033**	251	30	**1.41**	1.01	1.98	**0.044**	243	30
**Public wealth index (PWI)** ^††^	**0.28**	0.08	0.96	**0.043**	261	30	**0.37**	0.13	1.01	**0.053**	255	30	**0.44**	0.18	1.11	**0.081**	248	30
Crimes: drug trafficking^†^	0.94	0.69	1.29	0.721	268	30	1.01	0.78	1.30	0.950	297	30	1.06	0.87	1.29	0.581	296	30
New clients entering treatment^†^	0.85	0.70	1.03	0.105	253	28	0.87	0.72	1.04	0.116	252	28	0.88	0.75	1.04	0.128	246	28
Opioid Substitution Treatment (OST) clients ^†^	0.40	0.02	7.23	0.536	206	28	0.22	0.01	6.83	0.384	188	28	0.17	0.00	12.00	0.412	168	28
Syringes distributed or exchanged	0.88	0.61	1.26	0.482	158	26	0.97	0.83	1.13	0.657	136	26	0.97	0.80	1.17	0.744	113	26
Opioid injectors ^†^	0.96	0.74	1.23	0.719	166	29	0.93	0.75	1.14	0.481	138	29	0.85	0.51	1.43	0.542	110	26
Cocaine injectors ^†^	0.70	0.24	2.02	0.508	162	29	0.66	0.19	2.27	0.511	137	29	0.36	0.04	3.44	0.374	110	26
Total injectors ^†^	0.94	0.78	1.14	0.539	150	29	0.92	0.73	1.15	0.455	129	29	0.81	0.51	1.31	0.400	106	26
Daily opioid use	1.07	0.92	1.24	0.390	155	29	1.09	0.90	1.32	0.372	129	29	1.03	0.89	1.19	0.660	101	24

The dependent variable was dichotomous taking value 1 for years in which a European Economic Area (EEA) country was experiencing an HIV outbreak, 0 otherwise. The results include Odds Ratios (OR), Lower (L) and Upper (U) limits of the confidence interval (CI), P-values, the number of Observations (Obs) in each model, and the number of countries (C) from which data were obtained for at least one year.

^†^Per capita or per population

^††^PWI: Public Wealth Index = GDP per capita divided by S80/S20 ratio.

### Multivariable analyses

Table 4 shows the results of multivariable analyses that included the GDP growth rate in all models along with one of the Gini coefficient, S80/S20 ratio and proportion of people at risk for poverty one at a time. These variables were highly correlated and were not simultaneously included in multivariable models (Spearman’s coefficients: Gini and S80/S20, 0.98; Gini and Poverty, 0.86; S80/S20 and Poverty, 0.90).

**Table 4:** Multivariable logistic regression models.

	LAG 1 (1 year before current observation)						LAG 2 (2 years before current observation)						LAG 3 (3 years before current observation)					
		95% CI						95% CI						95% CI				
	OR	L	U	*P*	Obs.	C	OR	L	U	*P*	Obs.	C	OR	L	U	*P*	Obs.	C
***Models 1–3***																		
GDP growth rate	0.84	0.66	1.07	0.152	259	30	**0.72**	0.53	0.98	**0.039**	253	30	0.86	0.67	1.10	0.223	244	30
Population at risk of poverty	1.75	0.99	3.10	0.056			1.57	0.95	2.59	0.078			1.65	1.00	2.74	0.051		
***Models 4–6***																		
GDP growth rate	0.81	0.65	1.01	0.067	261	30	**0.68**	0.50	0.92	**0.012**	255	30	0.83	0.65	1.06	0.144	247	30
S80/S20 ratio	**2.95**	1.00	8.68	**0.050**			3.32	0.98	11.21	0.053			**3.89**	1.15	13.13	**0.029**		
***Models 7–9***																		
GDP growth rate	**0.80**	0.64	1.00	**0.053**	258	30	**0.65**	0.48	0.86	**0.003**	251	30	0.82	0.65	1.05	0.114	242	30
Gini coefficient	1.40	1.01	1.95	0.045			1.49	1.00	2.22	0.053			**1.43**	1.00	2.03	**0.048**		

The dependent variable was dichotomous taking value 1 for years in which a European Economic Area (EEA) country was experiencing an HIV outbreak, 0 otherwise. The independent covariates include the growth rate of Gross Domestic Product (GDP) in all models along with one of the Gini coefficient, S80/S20 ratio, and proportion of people at risk for poverty one at a time. The results include Odds Ratios (OR), Lower (L) and Upper (U) limits of the confidence interval (CI), P-values, the number of Observations (Obs) in each model, and the number of countries (C) from which data were obtained for at least one year.

Using 2-year lagged values, the GDP-related estimates adjusted for the percentage of the population that is at risk for poverty, for the S80/S20 ratio and the Gini coefficient were 0.72 (95% CI: 0.53 to 0.98), 0.68 (95% CI: 0.50 to 0.92), and 0.65 (95% CI: 0.48 to 0.86), respectively. Both of the income inequality measures retained, for all lagged values, their statistical significance (or were marginally non-significant at 0.05) when the analysis controlled for GDP change. The S80/S20 ratio that was estimated three years before the current observation was associated, when adjusted for GDP growth rate, with a nearly 3 times increase in the likelihood of an HIV outbreak among PWID in EEA countries (OR: 3.89, 95% CI: 1.15 to 13.13). One point increases of Gini index (towards more inequality) calculated one, two and three years before the current observation were associated with 40% (95% CI of OR: 1.01 to 1.95), 49% (95% CI of OR: 1.00 to 2.22) and 43% (95% CI of OR: 1.00 to 2.03) increase in the odds of an HIV outbreak, respectively.

## Discussion

Despite the decreasing trends in many European countries, Greece and Romania experienced a rapid spread of HIV among PWID in 2011, while in Bulgaria the reported rate of HIV infection in this population has been generally increasing since 2006. We carried out an ecological analysis to explore the association of macro-economic, policy and injecting risk variables with the increases in HIV diagnoses among people who inject drugs in European countries during a period of economic upheaval. Our analysis shows that, adjusted for GDP changes, inequalities in national income distribution are associated with the probability of an HIV outbreak among drug injectors in EEA countries during the last years. It also shows that GDP growth is associated with reduced probability of an outbreak.

Despite being a suboptimal indicator of a country’s wealth expansion and not a measure of personal income or living standards,[24] increasing GDP has been associated with outcomes such as longer life expectancy.[25] Conversely, declining GDP can act as a crude indicator of economic or social dynamics and changes related to adverse health outcomes, including HIV transmission in marginalized population groups. A recent longitudinal analysis investigated the relationship between the annual growth rate of GDP and a range of social and epidemiological outcomes in PWID in Greece.[21] The study revealed significant negative associations between the yearly change in GDP and the number of newly reported HIV infections among drug injectors.

There is evidence that, in general, wealthier people are probably healthier at the individual level,[26] but health is also affected by the overall income gap in the society in which individuals live. Improvements in national income distribution have been associated with longer life expectancy, reduced infant mortality, and fewer homicides.[27] The unequal distribution of national income has been related to higher prevalence rates of mental illness in rich countries, which, in turn, can promote unhealthy behaviors including illegal drug use.[28] In terms of infectious diseases, a recent analysis focused on the relationships between tuberculosis mortality rates and GDP per capita, poverty rates, and the Gini coefficient in 22 Latin American countries.[29] Increases in GDP had substantial positive impact on tuberculosis mortality (reduced) but when inequality was rising, greater GDP had no effect. Another cross-national study correlated wealth distribution and tuberculosis in Europe, and found a strong inverse relationship between the public wealth index and tuberculosis rates.[22] In our analysis, the same index was also strongly and inversely correlated with the odds of a significant increase in the number of HIV diagnoses among PWID in European countries. Using a broader sample of 90 countries, researchers have also examined the association between income inequality and HIV prevalence directly. Adjusting for economic development, cross-sectional regressions showed that there was a clear correlation between the Gini coefficient and the logarithm of HIV prevalence.[30] A study focusing on African countries found similar associations between the Gini coefficient and the logit function of HIV prevalence.[31] Another study involving 77 large metropolitan areas in the United States (US) [16] showed that income inequality was a predictor of both HIV prevalence and HIV incidence among PWID. Subsequent study of large US metropolitan areas found that income inequality was associated with higher mortality rates among heterosexuals living with Acquired Immune Deficiency Syndrome (AIDS).[32]

Wars, transitions, economic collapse, and ecological catastrophes (“Big Events”) can affect economic parameters including GDP and national income inequality. Friedman and colleagues [7] have discussed on how the political transition in former Soviet Union during the 1980s and 1990s was followed by economic instability and deepening poverty, and also by changes in the effectiveness of normative regulation and by youth alienation creating an HIV risk environment characterized by alcohol consumption, injecting drug use, and sexual risky behaviours leading to a large-scale epidemic. The theory of “Big Events” has not been fully formulated or tested. However, there are indications of its validity. For example, homelessness was strongly associated with HIV prevalence among PWIDs in a large respondent-driven sampling study (ARISTOTLE) that has been conducted in Athens during the crisis years.[33] Lack of stable accomodation was probably the result of economic hardship and may represent an intermediate cause of HIV transmission in PWIDs with declining GDP rate and increasing inequality as distal causes.

This analysis is subject to several limitations. Firstly, it is ecological in design. Of course, ecological research can analyze national-level data and trends, reveal population-level associations and processes that would not be observable or modifiable at individual level, and generate hypotheses. However, the results should be interpreted with caution given the caveats of measurement errors, omission of important predictors due to data not being available, and uncertain direction of causality. Population-level effects are not necessarily reducible to individual changes and, in addition, the pathways through which macro-level changes such as GDP decline and more unequal income distribution in “Big Events” situations affect HIV risk (if they do so) remain unknown, unmeasured or poorly understood, despite having received more research attention during the last decade.[7] Secondly, our analysis focused on reporting rates of HIV infection rather than on incidence estimates for HIV among PWID. Reliable estimates of HIV incidence among PWID are unavailable for the majority of EEA countries. It is important to note that reporting rates of newly diagnosed HIV infections depend on patterns of HIV testing and reporting, and may not be an adequate proxy for incidence. Nevertheless, molecular research has shown that in at least one of the countries with an ongoing HIV outbreak (Greece), the rapid HIV spread among injecting drug users started recently.[3,21] As an alternative, we could have used the available estimates of HIV prevalence among PWID. However, these were unavailable at national level for some countries and some years. Thirdly, in many European countries, HIV epidemics are localized geographically and analyses at national level may mask local or regional processes. However, local or regional analyses would definitely suffer from the substantial variability in data collection and estimation methods within and between countries. Fourthly, because of lack of data, we could not evaluate the potential role of key behavioural determinants of HIV risk in PWID such as receptive sharing of injecting equipment, unprotected sex, HIV testing uptake, knowledge of HIV status, and compliance with antiretroviral treatment. There is also a lack of data on additional factors associated with HIV infection risk among PWID such as population mobility, drug-trafficking, and OST and NSP coverage. Finally, the confidence intervals around the national inequality measures, especially the S80/S20 ratio, were large. The small number of outbreaks and the smaller sample size for the analyses including indices of national income inequality can explain the reduced level of precision.

Despite its limitations, the findings of our exploratory study may contribute to our understanding of HIV transmission in PWID, especially during times of economic hardships. Although the causal pathway to HIV epidemics among PWID is not fully understood, what national income inequality and low GDP growth rates probably do is to increase the vulnerability to an HIV outbreak in the population of drug injectors and thus the odds that in a given period such an outbreak will occur. According to our results, recovery of GDP growth alone may not necessarily lead to positive outcomes; if the generated wealth is unequally distributed, it may not reach marginalized sectors of society and thus may not improve health. It seems that redistributive measures that actively aim at reducing the gap between the richest and the poorest sections of the population in a country may help preventing adverse social and health effects. With respect to PWID, in combination with already extensively discussed measures to help to prevent or to respond to HIV epidemics in this group [18,19], cost-effective policy interventions that explicitly aim at improving access to social security benefits and treatment, and subsequently—through social reintegration programs—at getting a job and obtaining sufficient income may be crucial in averting HIV epidemics in this population.

## Supporting Information

S1 Dataset Data file. (XLS)

S1 Table Log-linear regression models for each country, with HIV rate as dependent variable and year (2003–2012) as independent variable. The results include regression coefficients (Coef.), Lower (L) and Upper (U) limits of the confidence interval (CI), P-values, and the number of Observations (Obs) in each model.
(DOC)
