# Supplementary material for: National Income Inequality and Declining GDP Growth Rates Are Associated with Increases in HIV Diagnoses among People Who Inject Drugs in Europe: A Panel Data Analysis
Source: PLoS One. 2015 Apr 15;10(4):e0122367. doi: 10.1371/journal.pone.0122367 (PMC4398461; doi:10.1371/journal.pone.0122367)
Supplement: S1 Table — The results include regression coefficients (Coef.), Lower (L) and Upper (U) limits of the confidence interval (CI), P-values, and the number of Observations (Obs) in each model. (DOC) [file pone.0122367.s002.doc]

**S1 Table**

Log-linear regression models for each country, with HIV rate as dependent variable and year (2003-2012) as independent variable. The results include regression coefficients (Coef.), Lower (L) and Upper (U) limits of the confidence interval (CI), P-values, and the number of Observations (Obs) in each model.

|  | **Coef.** | **95% CI** | | ***P*** | **Obs.** |
| --- | --- | --- | --- | --- | --- |
| **Country** |  | **L** | **U** |  |  |
| Austria | -0.085 | -0.151 | -0.019 | 0.018 | 9 |
| Belgium | -0.154 | -0.254 | -0.054 | 0.008 | 10 |
| Bulgaria | 0.231 | 0.066 | 0.397 | 0.013 | 9 |
| Croatia | -0.081 | -0.196 | 0.034 | 0.139 | 9 |
| Cyprus | 0.032 | -3.219 | 3.282 | 0.921 | 3 |
| Czech Republic | 0.033 | -0.071 | 0.137 | 0.484 | 10 |
| Denmark | -0.078 | -0.149 | -0.006 | 0.037 | 10 |
| Estonia | -0.194 | -0.302 | -0.086 | 0.003 | 10 |
| Finland | -0.096 | -0.164 | -0.027 | 0.012 | 10 |
| France | -0.100 | -0.125 | -0.076 | <0.001 | 10 |
| Germany | -0.067 | -0.103 | -0.031 | 0.003 | 10 |
| Greece | 0.355 | 0.095 | 0.615 | 0.014 | 10 |
| Hungary | 0.136 | -0.111 | 0.383 | 0.179 | 5 |
| Iceland | 0.253 | 0.003 | 0.502 | 0.048 | 7 |
| Ireland | -0.201 | -0.259 | -0.142 | <0.001 | 10 |
| Italy | -0.146 | -0.216 | -0.075 | 0.002 | 9 |
| Latvia | -0.078 | -0.133 | -0.023 | 0.012 | 10 |
| Lithuania | 0.004 | -0.079 | 0.088 | 0.910 | 10 |
| Luxembourg | -0.070 | -0.269 | 0.129 | 0.424 | 8 |
| Malta | 0.167 | -2.592 | 2.926 | 0.583 | 3 |
| Netherlands | -0.163 | -0.228 | -0.099 | 0.000 | 10 |
| Norway | -0.047 | -0.114 | 0.020 | 0.144 | 10 |
| Poland | -0.197 | -0.279 | -0.116 | 0.001 | 9 |
| Portugal | -0.258 | -0.326 | -0.190 | 0.000 | 10 |
| Romania | 0.435 | 0.138 | 0.733 | 0.011 | 9 |
| Slovakia | -0.015 | -0.256 | 0.225 | 0.875 | 7 |
| Slovenia^†^ |  |  |  |  | 7 |
| Spain | -0.162 | -0.185 | -0.140 | <0.001 | 10 |
| Sweden | -0.077 | -0.177 | 0.023 | 0.115 | 10 |
| United Kingdom | -0.048 | -0.080 | -0.016 | 0.009 | 10 |
| *Notes.*  † Multiple zero rates | | | | | |
